# Guanxinkang Decoction Exerts Its Antiatherosclerotic Effect Partly through Inhibiting the Endoplasmic Reticulum Stress

**DOI:** 10.1155/2014/465640

**Published:** 2014-05-18

**Authors:** Hao Wang, Zhi-Min Zheng, Bu-Lang Gao

**Affiliations:** ^1^Department of Traditional Chinese Medicine, The First Hospital of Shijiazhuang City, 36 Fanxi Road, Shijiazhuang, Hebei 050011, China; ^2^First Hospital of Shijiazhuang and Organ Transplantation Committee in Chinese Medical Association, 36 Fanxi Road, Shijiazhuang, Hebei 050011, China

## Abstract

*Purpose*. To investigate the antiatherosclerotic effect of Guanxinkang (GXK) decoction on the apoptosis, mitochondrial membrane potential (MMP), and endoplasmic reticulum stress (ERS) of human umbilical vein endothelial cells (HUVEC) pretreated with homocysteinemia (HCY). *Materials and Methods*. HUVEC were randomly divided into 5 groups: (1) blank control group (control), (2) model control group (model), (3) GXK low dose group, (4) GXK medium dose group, and (5) GXK high dose group. For the three GXK groups, HCY was given to reach the concentration of 3.0 mmol/L after HUVEC had been incubated with rabbit serum containing GXK for two hours. At 3, 6, 12, and 24 h after HCY had been incubated with the cells, the HUVEC were collected for test of the apoptosis rate, MMP, and GRP78 protein (reflecting ERS). *Results*. In the model control group, the apoptosis rate and GRP 78 protein expression of HUVEC significantly increased (*P* < 0.05), while MMP significantly decreased (*P* < 0.05) compared with the blank control group. After GXK treatment of medium and high doses, the apoptosis rate and the GRP 78 protein expression significantly (*P* < 0.05) decreased, while MMP significantly increased (*P* < 0.05) in a time-dependent manner compared with the model control group. *Conclusion*. GXK can antagonize the injury of HUVEC caused by HCY and the antagonism effect increases with the concentration and treatment duration of GXK, with the possible mechanism of GXK antagonism being through inhibiting ERS caused by HCY.

## 1. Introduction

Atherosclerosis is a chronic inflammatory condition in which the arterial wall thickens because of the accumulation of cholesterol, macrophages, and smooth muscle cells (SMC), ultimately restricting blood flow through the artery. It is the primary pathologic condition underlying coronary artery and cerebrovascular diseases resulting in heart attack and stroke, respectively. Prevention of atherosclerosis can reduce the incidence and mortality of cerebrovascular and cardiovascular diseases.

Hyperhomocysteinemia is an independent risk factor for atherosclerotic cardiovascular disease, stroke, and peripheral vascular disorders [1, 2] because it is a potent proinflammatory factor to accelerate the development of atherosclerosis [3]. Epidemiological studies in the general population have indicated a positive correlation between an elevation of total plasma homocysteinemia (HCY) and stroke, myocardial infarction, peripheral vascular disease, and venous thromboembolism [4]. HCY is a thiol-containing amino acid formed during the intracellular demethylation of methionine and causes multifold effects through the reactivity of its sulfhydryl group. Increase in levels of HCY impairs cellular function and causes loss of endothelial antithrombotic function, lipid peroxidation, impairment of platelet aggregation, enhanced oxidative stress, and apoptosis [5–7]. These unfavorable vascular effects of HCY are caused by the generation of reactive oxygen species (ROS) [8], which play a critical role in the damage and dysfunction of the endothelium [9]. ROS acts as an upstream factor for mitochondrial membrane depolarization, and increased production of ROS is implicated in loss of mitochondrial membrane potential (MMP) [10] and induction of members of Bcl2 protein family. Mitochondria release cytochrome-c and caspases that result in eventual endothelial cell apoptosis an cardiac dysfunction [11].

The endoplasmic reticulum (ER) is a multifunctional intracellular organelle responsible for the synthesis and folding of proteins as well as calcium storage and signaling. When its function is disturbed by various physiological and pathological conditions like misfolded protein accumulation, hypoxia, Ca^2+^ depletion, or microbial infection, endoplasmic reticulum stress (ERS) develops, and ERS can regulate process such as cell survival and cell death [12]. Cells alleviate ERS through the unfolded protein response, and the upregulation of ER chaperones, such as the glucose-regulated protein 78 (GRP78), contributes to the repair of unfolded proteins. However, if stress is sustained, the unfolded protein response causes cell death by transcriptional induction of CCAAT/enhancer-binding protein homologous protein (CHOP), the caspase-12 dependent pathway, and activation of the c-Jun NH2-terminal kinase dependent pathway [13]. GRP78 is the major ER-resident chaperone and the most abundant glycoprotein in the ER. It is widely used as a biomarker of ERS because it plays a critical role in protein folding and ER Ca^2+^ binding [14].

HCY-induced vascular injury may also involve ERS and activation of the unfolded protein response caused by incorrect protein folding [15]. HCY can cause ERS by disrupting disulfide bond formation, causing misfolding of proteins traversing the ER. HCY induces the expression of GADD153 (a basic region leucine zipper transcription factor involved in ERS-induced cell death), the ER chaperones GRP78 and GRP94, and Herp (a protein involved in the degradation of misfolded ER protein) [16].

Guanxinkang decoction (GXK) is a compound traditional Chinese herbal medicine composed of the following six Chinese herbs of Radix Astragali,* Trichosanthes*, Radix Salviae Miltiorrhizae,* Allium macrostemon*,* Pinellia tuberifera*, and Radix Puerariae. This decoction has been proved to have an antiatherosclerotic effect and this effect is partly through its interference with lipid regulation, inflammation activities, and ion channels [17–20]. However, the exact effect of this decoction on the endothelial cells is not known, and this study intended to investigate its protection effect on the endothelial layer of the vascular wall by studying the apoptosis rate, the MMP, and the GRP78 expression levels of the human umbilical vein endothelial cells (HUVEC) treated with GXK and HCY in sequence.

## 2. Materials and Methods

### 2.1. Agents

Trypsin and Dulbecco's modified Eagle's medium (DMEM) were purchased from Gibco (Invitrogen, Shanghai, China), HCY and propidium iodide (PI) from Sigma-Aldrich (China branch), and MMP detection kit (JC-1) was from Beyotime Institute of Biotechnology (Shanghai). Goat anti-GRP78 monoclonal antibody was bought from Santa Cruse Corporation (USA), and HRP enzyme-labeled rabbit secondary antibody against goat IgG and Western blotting reagents were from Boster Biotech (Wuhan, China). HUVEC was from the central laboratory of the affiliated hospital to Liaoning University of Traditional Chinese Medicine.

### 2.2. Preparation of Rabbit Medicine Serum

This study protocol was approved by the animal care and ethics committee of our hospital. 30 New Zealand white rabbits were randomly divided into five groups used for the preparation of rabbit medicine serum: blank control group (control), model control group (model), GXK low dose group (GXKL), GXK medium dose group (GXKM), and GXK high dose group (GXKH). The lowest dosage of Guanxinkang of human is 10 g; according to the animal equivalent dose conversion, the dosage of the rabbit is 1.45 g/kg which was used in GXKL. Then it was increased 2 times and 4 times, respectively, in GXKM and GXKH, as follows: (1) blank control group (control): the rabbits were fed no HCY nor GXK so that the rabbit serum contained no HCY or GXK; (2) model control group (model): the rabbits were fed as the blank control group has been fed; (3) GXK low dose group (GXKL): GXK at 1.45 g/kg was used to feed the rabbits through gastric tube so that the rabbit serum contained low dose GXK; (4) GXK medium dose group (GXKM): GXK at 2.90 g/kg was used to feed the rabbits through gastric tube so that the rabbit serum contained medium dose GXK; (5) GXK high dose group (GXKH): GXK at 5.80 g/kg was used to feed the rabbits through gastric tube so that the rabbit serum contained high dose GXK. Seven days after the rabbits were treated with different medicines, the rabbits were anesthetized and blood was aspirated from the hearts. The blood was centrifuged for 10 min at 2000 r/m within one hour, and the supernatant was used as the rabbit serum which was further filtered and kept for experiment.

### 2.3. Treatment of HUVEC

The HUVEC were grown in the DMEM containing 20% fetal bovine serum in an incubator under 5% CO_2_. The cells used in this study were from passage 3 and the cells were washed twice with the DMEM and adjusted to the concentration of 1 × 10^5^/mL before experiment. The cells were randomly divided into 5 groups which were given 10% rabbit serum containing different medicines for treatment: (1) blank control group (control): 10% rabbit serum from the blank control group of rabbits was used with no HCY or GXK being added; (2) model control group (model): 10% rabbit serum from the model control group of rabbits was used; then HCY was given to reach the concentration of 3.0 mmol/L after the 10% blank control rabbit serum was added for two hours; (3) GXK low dose group (GXKL): 10% rabbit serum containing low dose of GXK was given; (4) GXK medium dose group (GXKM): 10% rabbit serum containing medium dose of GXK was given; (5) GXK high dose group: 10% rabbit serum containing high dose GXK was added. For the three low-high dose GXK groups, HCY was given to reach the concentration of 3.0 mmol/L after the HUVEC had been incubated with GXK at low, medium, and high doses for two hours. Then, the HUVEC were collected for test at 3, 6, 12, and 24 h after HCY had been incubated with the cells.

### 2.4. Flow Cytometry

The HUVEC in every group were centrifuged and collected at 3, 6, 12, and 24 h after incubation with HCY. The cells were washed twice with PBS and adjusted to 1 × 10^6^/mL in concentration. PI was added into every sample group which was put in a 4C° refrigerator for staining in the dark for 20–30 min. Then, PI was washed off, PBS was added, and the cells were screened using a 300-mesh sieve. Flow cytometry was performed to detect the DNA content and the apoptosis rate of cells. The Cell Quest software (FACSCalibur, BD, USA) was used to calculate the cell apoptosis rate.

### 2.5. Detection of MMP

The HUVEC in every group were centrifuged and collected at 3, 6, 12, and 24 h after incubation with HCY. The supernatant was discarded. DMEM of 0.5 mL (with no serum) was added into every tube of cells, and a positive control was set up. The CCCP (10 mM) provided in the MMP detection kit was diluted at 1 : 1000 and was added into the cell culture medium, and the medium was diluted to 10 *μ*M. The cells in the positive control group were added into the cell culture medium for incubation of 20 min. Then, one mL JC-1 dye was added into every tube of cells and blended completely. The cells were incubated in an incubator at 37C° for 20 min. Then, the supernatant was aspirated; the cells were washed twice with JC-1 dye. DMEM of 1 mL was added and flow cytometry was performed at the conditions of excitation 480 nm and emission 520 nm using Cell Quest software.

### 2.6. Western Blot Analysis for GRP78

Twenty-four hours after the HUVEC were treated with HCY, the culture medium was aspirated and the cells were scraped with a cytobrush. PBS was added into the cells for centrifugation, and culture flasks and the cytobrush were washed. The rinse solution together with the cells was put into the centrifugal tubes for centrifugation at 300 g for 5 min. Then, the cell precipitate was aspirated, washed twice with PBS, and added with cell lysate. The cells were scraped with a cytobrush and put into the Eppendorf tube. The cells were put on ice for 30 min for their complete degradation and then centrifuged at 4C° and 12000 g for 10 min. The supernatant was collected for the total protein. Then, Western blot analysis for GRP78 was performed as instructed.

### 2.7. Statistical Analysis

All the data were expressed as mean ± standard deviation using SPSS10.0 software for the analysis of two-factor ANOVA, with the significant *P* value set at <0.05.

## 3. Result

### 3.1. Cell Apoptosis

**Figure 1:**
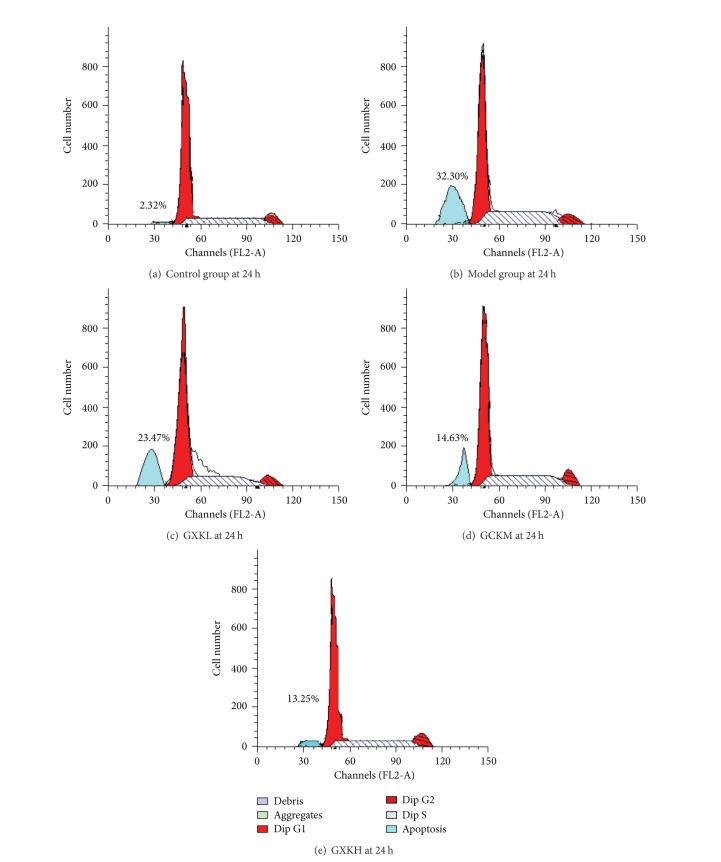
Apoptosis rate under the action of Guanxinkang at 24 h. (1) Compared with the control group, apoptosis rate of each group was increased, especially in the model group (*P* < 0.05). (2) Apoptosis rate of GXKL decreased not significantly. (3) Apoptosis rate of GXKM or GXKH decreased significantly. Compared with the model group, the difference is significant (*P* < 0.05).

Compared with the blank control group, the cell apoptosis rate was significantly (*P* < 0.05) increased in all the other groups especially in the model control group. However, the cell apoptosis rate in the groups treated with different concentrations of GXK was lower than in the model control group, and the difference was more significant with increase of treatment duration. The difference was the greatest at the treatment point of 24 h. At the same time point, the apoptosis rate decreased insignificantly (*P* > 0.05) in the GXKL group but significantly (*P* < 0.05) in both the GXKM and GXKH groups compared with the model group (Table 1 and Figure 1).

### 3.2. MMP Detection

**Figure 2:**
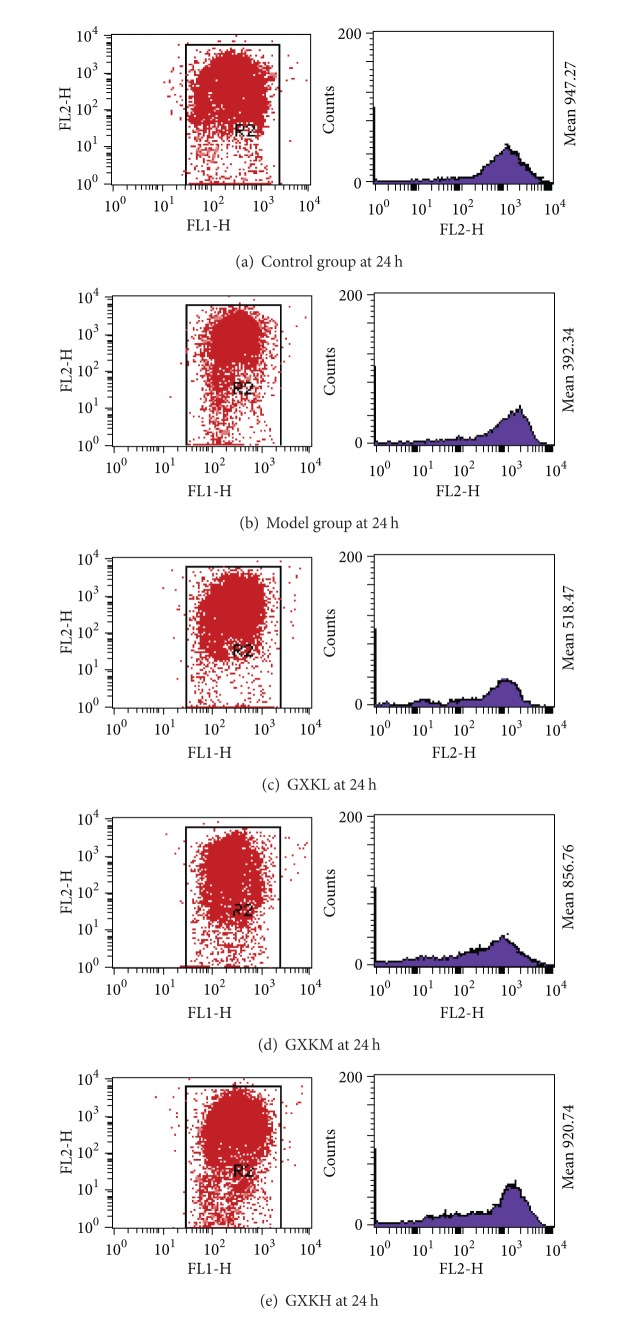
MMP of HUVEC under the action of Guanxinkang at 24 h. (1) Compared with the control group, the data of MMP in each group decreased, especially in the model group (*P* < 0.05). (2) The data of MMP in cells under the action of GXK of different doses was significantly higher than that in the model group, and with the effect time progressing, the difference is more obvious. (3) The data of MMP of GXKL increased not significantly. (4) The data of MMP of GXKM and GXKH increased significantly (*P* < 0.05).

Compared with the blank control group, the MMP decreased significantly (*P* < 0.05) in all the other groups especially in the model control group. However, the MMP in all the groups treated with different concentrations of GXK was higher than in the model control group, and the difference was more significant with increase of treatment duration. The difference was the greatest at the treatment point of 24 h. At the same time point, the MMP increased insignificantly (*P* > 0.05) in the GXL group but significantly (*P* < 0.05) in both the GXKM and GXKH groups compared with the model control group (Table 2 and Figure 2).

### 3.3. Expression of GRP78 in HUVEC

**Figure 3:**
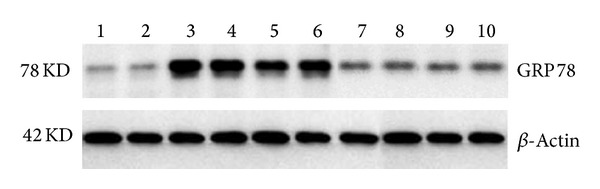
GRP78 of HUVEC at 24 h under the action of Guanxinkang. (1-2): blank control; (3-4): HCY model control; (5-6): Guanxinkang low dose group; (7-8): Guanxinkang medium dose group; (9-10): Guanxinkang high dose group. (1) GRP78 protein is more significantly expressed in the cells in model group (*P* < 0.05). (2) At 24 hours, expression of GRP78 in GXKL has not decreased obviously. (3) It increased significantly in GXKM and GXKH (*P* < 0.05).

Compared with the blank control group, the expression of GRP78 protein increased significantly (*P* < 0.05) and reached 0.36 ± 0.07 at 24 h after HCY treatment in the model control group (Table 3). The expression decreased in all the other groups with GXK treatment and the decrease became more apparent with increase of treatment duration. The decrease of the expression was the greatest at 24 h. At the same time point, the decrease of the expression of GRP78 protein was not significant (*P* > 0.05) in the GXKL group but significant (*P* < 0.05) in both the GXKL and GXKH groups compared with the model control group, with the best result being obtained at 24 h (0.11 ± 0.07) (Figure 3).

## 4. Discussion

In this study, we investigated the role of GXK decoction in protecting the endothelial cells and found that GXK can significantly antagonize the effect of HCY on HUVEC. With the treatment of GXK, the HUVEC apoptosis rate and the expression of GRP78 protein are significantly decreased, while the HUVEC MMP is increased, favoring a protection effect on the endothelial cells.

Atherosclerosis is the hardening and narrowing of the arteries and this progressive process silently and slowly blocks arteries, putting blood flow at risk. Atherosclerosis is the primary cause of heart attacks, strokes, and peripheral vascular diseases, which are usually called cardiovascular disease, the number one killer in USA, with more than 800,000 deaths in 2005 [21]. Hyperhomocysteinemia is an independent risk factor for atherosclerosis [1, 2] and can cause apoptosis and decrease of MP of endothelial cells. Tyagi et al. [7] studied the mitochondrial mechanism of microvascular endothelial cell apoptosis caused by hyperhomocysteinemia and found that HCY-mediated ROS production promotes endothelial cell apoptosis in part by disturbing MP (decrease and loss of MP), which results in subsequent release of cytochrome-c and activation of caspase-9 and caspase-3, leading to cell death. Hyperhomocysteinemia is a potent proinflammatory factor to accelerate the development of atherosclerosis [22]. Pathophysiological levels of HCY could interfere with human monocyte function by upregulating monocyte chemoattractant protein 1 and IL-8 expression and secretion via oxidative stress [23].

Endoplasmic reticulum contains many proteins, for example, PERK (PKR-like eukaryotic initiation factor 2*α* kinase), IRE-1 (inositol requiring enzyme-1), ATF6 (activating transcription factor 6), CHOP (C/EBP homologous protein), GRP78 (glucose-regulated protein 78) and so on. GRP78 protein is the major ER-resident chaperone and is widely used as a biomarker of ERS [24] because it plays a crucial role in protein folding and ER Ca^2+^ binding. It facilitates protein folding in the ER, and thus it reduces the number of misfolded proteins and alleviates ERS. A common stimulus for the induction of GRP78 is the presence of misfolded proteins in the ER15. Studies demonstrated that HCY induced vascular injury involving ERS and the increase of GRP78 protein [15, 16]. So only the expression of GRP78 was observed in this experiment.

In our study, HCY increased the apoptosis rate and GRP78 protein expression but decreased MP in the model control group. GXKL reduced apoptosis and MMP (*P* > 0.05) but showed no obvious effect on HCY-induced GRP78. This is because apoptosis can occur directly through the mitochondrial pathway and also can be induced by ER stress [25, 26]. The treatment of GXKM and GXKH significantly antagonized these effects caused by HCY. GXK has been proved to have a protective effect on the endothelial cells against atherosclerosis partly through its interference with lipid regulation, inflammation activities, and ion channels [17–20]. Our study demonstrated that GXK could also antagonize the damage effect HCY on the endothelial cells through inhibiting ERS by decreasing the apoptosis rate and the expression of GRP78 protein and increasing MP.

GXK is a compound traditional Chinese herbal medicine composed of six Chinese herbs of radix astragali,* Trichosanthes*, Radix Salviae Miltiorrhizae,* Allium macrostemon*,* Pinellia tuberifera*, and Radix Puerariae. The medicine can invigorate qi and straighten health so as to adjust the balance of Yin and Yang in viscera in order to cure the basic deficiency. At same time modern research shows that it eliminates phlegm and smoothens vessel, gets rid of pathogenic factors, smoothens main and collateral channels, and relieves symptoms. The formation of this compound medicine is based on the treatment theory of traditional Chinese medicine (TCM) which emphasizes the self-healing capacity of the human mind and body and focuses on the whole rather than on the parts, on disease prevention rather than on treatment, and on the diseased person rather than on the suffering disease. On the contrary, Western biomedicine usually focuses on the diseased parts of the body, on the treatment of disease, and on the suffering disease. Because of the limits of Western biomedicine nowadays in treating degenerative diseases, stress-related diseases, and most chronic diseases, more and more people began to lay their eyes on TCM [27–29]. Chinese herbs as raw materials of medicine are increasingly favored by people worldwide for their unique advantages in preventing and curing diseases, rehabilitation, and health care. More importantly, the unique theory of TCM provides some implications for renewing the treatment ideas in fight against cardiovascular diseases. This is why we conducted this study investigating the possible mechanism of GXK in preventing endothelial damage caused by HCY.

Based on domestic studies in China, the compound medicine of GXK has the function of improving the antioxidase activity of the body, eliminating free radicles, protecting the cells' ultrastructures especially the mitochondrial structure, decreasing the adherence rate of platelets, regulating blood lipids, and improving the blood rheology [30–34], thus having an effect against atherosclerosis. This study investigated the possible molecular mechanism of GXK on the prevention of atherosclerosis and confirmed the effect of GXK on the expression of the endoplasmic protein GRP78. Further study is needed to investigate the next pathway and signal transmission after GRP78 protein.

In conclusion, the traditional Chinese compound medicine GXK can antagonize the endothelial apoptosis caused by HCY and decrease the expression of the endoplasmic GRP78 protein, and the effect is in a positive proportional relationship with the concentration and action duration of GXK. This study indicates that GXK exerts its effects by inhibiting ERS and subsequent signal transmission of apoptosis.

## Figures and Tables

**Table 1:** Apoptosis rate under the action of GXK at different points ($\overset{-}{X}\pm s$, *n* = 3).

Groups	Apoptosis rate (%)			
	3 h	6 h	12 h	24 h
Control	2.12 ± 0.41	1.96 ± 0.49	2.03 ± 0.38	2.32 ± 0.67
Model	10.22 ± 1.43	18.74 ± 0.60*	24.83 ± 1.97*	32.15 ± 2.78*
GXKL	8.42 ± 1.14	14.26 ± 2.17	20.38 ± 3.10	23.51 ± 2.16^#^
GXKM	6.25 ± 0.95	8.46 ± 1.79^#^	9.81 ± 2.45^#^	15.34 ± 1.81^#^
GXKH	5.77 ± 1.09	6.74 ± 1.93^#^	7.92 ± 3.56^#^	11.35 ± 2.35^#^

Note: *compared with control group, *P* < 0.05; ^#^compared with model group, *P* < 0.05; GXK: Guanxinkang; GXKL: Guanxinkang low dose group; GXKM: Guanxinkang medium dose group; GXKH: Guanxinkang high dose group.

**Table 2:** MMP of HUVEC under the action of GXK at different times ($\overset{-}{X}\pm s$, *n* = 3).

Groups	MMP			
	3 h	6 h	12 h	24 h
Control	1085.21 ± 12.24	992.24 ± 13.32	965.67 ± 12.67	932.43 ± 15.14
Model	712.25 ± 16.31*	584.69 ± 19.21*	498.17 ± 20.26*	389.56 ± 22.36*
GXKL	737.79 ± 17.42	673.73 ± 16.91	624.29 ± 17.41	510.32 ± 20.47^#^
GXKM	755.22 ± 17.21	736.53 ± 17.69^#^	799.43 ± 16.38^#^	856.26 ± 15.81^#^
GXKH	781.27 ± 16.31	801.25 ± 16.31^#^	863.64 ± 14.93^#^	915.64 ± 16.83^#^

Note: GXK: Guanxinkang; GXKL: Guanxinkang low dose group; GXKM: Guanxinkang medium dose group; GXKH: Guanxinkang high dose group; MMP: mitochondrial membrane potential; *compared with control group, *P* < 0.05; ^#^compared with HCY model group, *P* < 0.05.

**Table 3:** Level of protein GRP78 in HUVEC under the action of GXK at different points ($\overset{-}{X}\pm s$, *n* = 3).

Groups	Mean optic density			
	3 h	6 h	12 h	24 h
Control	0.06 ± 0.01	0.07 ± 0.02	0.07 ± 0.01	0.08 ± 0.03
Model	0.19 ± 0.06	0.24 ± 0.05*	0.27 ± 0.09*	0.36 ± 0.07*
GXKL	0.16 ± 0.04	0.21 ± 0.07	0.20 ± 0.04	0.25 ± 0.02
GXKM	0.15 ± 0.05	0.17 ± 0.02^#^	0.16 ± 0.06^#^	0.13 ± 0.03^#^
GXKH	0.14 ± 0.03^#^	0.18 ± 0.08^#^	0.14 ± 0.09^#^	0.11 ± 0.07^#^

Note: *compared with control group, *P* < 0.05; ^#^compared with HCY model group, *P* < 0.05; GXK: Guanxinkang; GXKL: Guanxinkang low dose group; GXKM: Guanxinkang medium dose group; GXKH: Guanxinkang high dose group.
